# A Systematic Review on the Effectiveness of Antipsychotic Drugs on the Quality of Life of Patients with Schizophrenia

**DOI:** 10.3390/brainsci13111577

**Published:** 2023-11-10

**Authors:** Gaia Sampogna, Matteo Di Vincenzo, Luigi Giuliani, Giulia Menculini, Emiliana Mancuso, Eleonora Arsenio, Salvatore Cipolla, Bianca Della Rocca, Vassilis Martiadis, Maria Salvina Signorelli, Andrea Fiorillo

**Affiliations:** 1 Department of Psychiatry, University of Campania “L. Vanvitelli”, 80138 Naples, Italyluigi.giuliani.91@gmail.com (L.G.); salvatore2211@gmail.com (S.C.);; 2 Department of Psychiatry, University of Perugia, 06132 Perugia, Italy; 3 Department of Mental Health, ASL Napoli 1 Centro, 80147 Naples, Italy; 4 Department of Psychiatry, University of Catania, 95124 Catania, Italy

**Keywords:** schizophrenia, quality of life, antipsychotic drugs, personalized treatment, neuroleptics, outcome assessment

## Abstract

Pharmacological antipsychotic drug interventions represent the cornerstone of the management of patients with schizophrenia and other psychotic spectrum disorders. The choice of the “best” treatment should be made on the basis of several clinical domains. However, despite available treatments, the quality of life reported by patients with schizophrenia taking antipsychotics is still very poor, and this outcome is rarely taken into account in trials assessing the efficacy and effectiveness of antipsychotic treatments. Therefore, we performed a systematic review in order to assess the impact of antipsychotic treatment on patients’ quality of life. In particular, we aimed to identify any differences in the improvement in quality of life according to the (a) type of formulation of antipsychotic drugs (i.e., oral vs. depot vs. long-acting injectable); (b) type of the drug (first vs. second vs. third generation); and (c) patients’ clinical characteristics. One hundred and eleven papers were included in the review. The main findings were as follows: (1) quality of life is usually considered a secondary outcome in trials on the efficacy and effectiveness of drugs; (2) second-generation antipsychotics have a more positive effect on quality of life; and (3) long-acting injectable antipsychotics are associated with a more stable improvement in quality of life and with a good safety and tolerability profile. Our systematic review confirms that quality of life represents a central element for selecting the appropriate treatment for people with schizophrenia. In particular, the availability of new treatments with a better tolerability profile, a proven effectiveness on patients’ cognitive and social functioning, and with a more stable blood concentration might represent the appropriate strategy for improving the quality of life of people with schizophrenia.

## 1. Introduction

Schizophrenia is a severe mental disorder, with a prevalence rate of 1 in 300 people (0.32%) worldwide and with approximately 24 million people suffering from this disorder. Schizophrenia is associated with high levels of personal and social burden [1,2]. Patients suffering from schizophrenia are at higher risk—compared to the general population—of developing physical comorbidities, including cardiovascular diseases, diabetes, obesity, and cancer, with a significant reduction in life expectancy by 15–20 years [3]. Furthermore, patients with schizophrenia often suffer from comorbid mental disorders, including anxiety disorders, with a prevalence rate of 45% [4]; personality disorders, with a prevalence rate of 25% [5]; or substance use disorders, in up to 70% of patients with schizophrenia [6]. Low levels of long-term functional recovery have been reported by patients with schizophrenia, with frequent relapses and voluntary/involuntary hospitalizations. In fact, the disorder often presents a recurrent course, with relapses and requiring a long-term pharmacological treatment [7,8]. Schizophrenia has been conceptualized as a heterogeneous disorder with a complex etiopathogenesis, caused by the interplay between genetic, environmental, and psychosocial factors [9,10]. Thus, multilevel, integrated, and personalized treatment is essential for people suffering from schizophrenia [7,11,12]. The treatment choice should be made on the basis of several salient clinical domains, such as positive and negative symptom dimensions [13,14,15], other psychopathological components, type of onset and course, neurocognition and social cognition, neurodevelopmental indicators, social functioning, quality of life and unmet needs, clinical staging, antecedent and concomitant psychiatric conditions, physical comorbidities, family history, history of obstetric complications, early and recent environmental exposures, protective factors and resilience, and internalized stigma [16,17].

Remission and recovery rates in people with schizophrenia are still not satisfactory and several unmet clinical needs are reported, including the persistence of negative and cognitive symptoms, and the high relapse and mortality rates in those patients [18,19]. Due to the persistence of residual symptoms, patients’ functioning can be persistently impaired, affecting the achievement of full-functional recovery [20].

The treatment plan includes both pharmacological and non-pharmacological interventions, usually proposed to the patient according to a sequential process [21,22,23]. In particular, pharmacological drug interventions represent the cornerstone in the management of schizophrenia and other psychotic spectrum disorders [24,25,26]. People treated with antipsychotics reported a lower mortality rate [3,27] compared to non-treated patients.

However, the levels of quality of life reported by patients taking antipsychotics are still very poor [28,29], and this outcome is rarely taken into account in trials assessing the efficacy and effectiveness of antipsychotic treatments [30]. In particular, patients’ quality of life is mainly influenced by the persistence of specific symptom domains, such as negative symptoms [31], and by the presence of physical comorbidities [32,33]. Therefore, evaluating the impact of antipsychotic treatment on quality of life is essential in order to achieve patients’ full recovery.

We performed a systematic review of the available literature in order to assess the impact of antipsychotic treatment on the levels of quality of life. In particular, we aimed to identify any differences in the improvement in quality of life according to the (a) type of formulation of antipsychotic drugs (i.e., oral vs. depot vs. long-acting injectable); (b) type of the drug (first vs. second vs. third generation); and (c) patients’ clinical characteristics.

## 2. Materials and Methods

**Figure 1:**
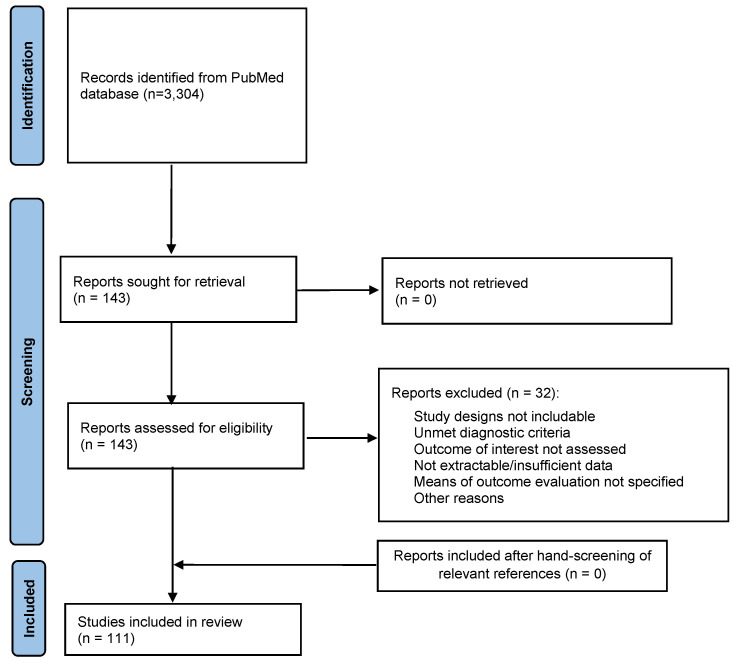
PRISMA flowchart.

This review was performed in five stages: definition of the problem, literature search, data evaluation, data analysis, and presentation of findings. The identification of the research question was performed by using the PICO (patients, intervention, comparison, outcome) framework. In particular, studies including adult (aged 18 or more) inpatients or outpatients with schizophrenia or psychotic disorders, treated with antipsychotic medication, and providing data on quality of life, personal functioning, and/or satisfaction with antipsychotic treatments were included in the review. The search terms “(antipsychotic agents [MeSH] OR antipsychotic drugs OR antipsychotic medications OR neuroleptic drugs OR antipsychotics OR neuroleptics OR long-acting injectable) AND (quality of life [MeSH] OR life quality health-related quality of life OR HRQoL)” were entered into ERIC, MEDLINE, WebofScience, PsycARTICLES, PsycINFO, SCOPUS, and PUBMED (Figure 1). Terms and databases were combined using the Boolean search technique, which consists of a logical information retrieval system (two or more terms combined to make searches more restrictive or detailed).

In this review, case–controls, cohorts, randomized control trials (RCT), as well as retrospective and prospective real-world experience studies were included. Publications were identified by searching electronic databases and the reference lists of selected articles. The search was limited to studies published in English and was conducted starting from inception to 30 April 2023.

Studies in which psychiatric diagnoses were performed according to the current nosographic criteria (DSM-5 or ICD-11, or previous versions according to the period of time when the study was carried out) and in which measures of quality of life (e.g., self- and clinician-administered scales) were clearly used were considered eligible.

Case reports, editorials, letters to the editor, and reviews were excluded. However, the reference lists of reviews were searched in order to identify relevant primary publications. Similarly, studies reporting only a hypothesis without original data obtained from quality-of-life measures were not considered eligible. Studies on underage children and/or adolescents were excluded since quality of life was assessed using different tools. Grey literature was considered, if sufficient information was provided.

The review process was performed in accordance with PRISMA guidelines, and a PRISMA flowchart was included (Figure 1).

### Study Selection and Data Extraction

Three independent researchers (MDV, GM, and LG), having a wide expertise in the field of psychiatry research, including the preparation of systematic reviews, performed literature search, title-abstract screening, and full-text screening. References of relevant articles were hand-searched to evaluate further papers of potential interest. The search process was carried out using a double-blind methodology and discrepancies were solved through consensus. A further researcher (GS) was consulted if needed. The independent researchers also performed data extraction from the included papers, by using an ad hoc extraction tool where the following information was collected: author(s) and year, country, study design, setting, study sample, diagnostic assessment, pharmacological treatment, study outcomes, means of quality-of-life assessment, and relevant results concerning quality of life.

Authors screened the articles identified by the searches and then performed a full-text review of those that appeared relevant to the research topic based on titles and abstracts. Disagreements that arose between the reviewers were resolved through discussion, and in the case of continued disagreement, with the assistance of a senior researcher (AF).

Risk of Bias evaluation has been reported as Supplementary Information Tables.

## 3. Results

A total of 3304 records were initially identified, and after duplicate removal, 3123 were assessed by reading abstracts. Following this step, 1256 were excluded. Therefore, 1867 full articles were analyzed and 110 were included in the review (Figure 1).

The main findings of the included papers are summarized in Table 1, Table 2, Table 3, Table 4 and Table 5.

A total of 54% of papers (N = 60) assessed the association between oral antipsychotics and quality of life, whereas 31% (N = 34) of papers assessed the association between long-acting injection (LAI) and quality of life (Table 2). In 10% of papers (N = 11), no data were available on the specific type of pharmacological agent included in the study [34,35,36,37,38,39,40,41,42,43,44]; in 4% of papers, a combination of oral and LAI treatments was analyzed, while in the remaining papers, a polytherapy was reported [45,46] (Table 4). The sample size of the included studies significantly varied, ranging from seven subjects with a first episode of psychosis in Cervone et al. [47] to 16,091 patients suffering from schizophrenia in Adrianzén et al. [48].

Longitudinal or cross-sectional observational studies as well as randomized controlled trials represent the most common designs.

In 55 studies, patients were recruited in an outpatient setting, while in 31 studies, patients were recruited at both inpatient and outpatient units. In 14 studies, no clear information was given on the setting of recruitment.

In the vast majority of studies, included patients were suffering from schizophrenia or another schizophrenia spectrum disorder (schizoaffective disorder, delusional disorder, schizophreniform disorder, brief psychotic disorder), while seven studies [47,49,50,51,52,53,54,55] included patients with a first episode of psychosis (FEP). Lambert et al. [56], Tsang et al. [57], Aitchison et al. [58], and Vrbova et al. [43] used the broad inclusion criterion of “having a psychotic disorder”.

The diagnosis was established through the different editions of the Diagnostic and Statistical Manual of Mental Disorders [59,60,61,62] and the International Classification of Disease [63,64], and was confirmed using validated assessment tools, such as the Positive and Negative Syndrome Scale (PANSS) [65], the Brief Psychiatric Rating Scale (BPRS) [66], the Clinical Global Impression (CGI) [67], and the Global Assessment of Functioning [68].

Quality of life was assessed using different validated and reliable tools, including the Quality-of-Life Scale (QLS); the WHO’s WHOQOL-BREF; the Short-Form Health Survey-36 (SF-36); and the Short-Form Health Survey-12 (SF-12). The QLS [69] is a semi-structured interview aimed at rating the deficit derived from schizophrenia by exploring social relations, roles at home/work/school, motivation, and daily activities. The WHOQOL-BREF [70] is a 26-item self-reported tool, focusing on the personal perception of quality of life in terms of physical, psychological, social, and environmental aspects. Health-related quality of life is measured by using the self-reported SF-12 [71] and SF-36 [72], which include perceived levels of physical and social limitations, pain, and energy.

The paper by Meltzer et al. [73] was the first study including a specific focus on the association between the use of antipsychotic medications and quality of life in a small sample (N = 38) of patients with a diagnosis of schizophrenia according to the DSM-III-R.

Among the most recently published papers, several papers have shown favorable effects on the quality of life of aripiprazole, blonanserin, paliperidone [74], lurasidone [75], and long-acting atypical antipsychotics [76,77].

A cohort study by Lorenzo et al. [78] did not detect significant differences at the WHOQOL-BREF total and subscale scores between haloperidol decanoate and paliperidone palmitate (monthly and quarterly injections). Nasrallah et al. [77] did not find any difference in quality of life between paliperidone palmitate and aripiprazole lauroxil.

The majority of studies included second- or third-generation antipsychotic medications. In particular, 29 studies (26.1%) analyzed first-generation antipsychotics (FGAs), with haloperidol being the most studied drug [53,79,80,81,82,83,84,85,86], while flupentixol was the most studied among first-generation depot antipsychotics [50,51,55,87]. The vast majority of papers (71.8%, 79 out of 110 papers) on the efficacy of second-generation antipsychotics (SGAs) included the assessment of quality of life as an outcome measure. In particular, olanzapine, risperidone, and quetiapine were the most studied oral drugs, while risperidone LAI and paliperidone palmitate were the most studied ones when considering injectable formulations. The impact of clozapine on quality of life was investigated in 13 studies [52,73,83,88,89,90,91,92,93,94,95,96,97].

Third-generation antipsychotics (TGAs) included aripiprazole (both oral and LAI formulation), cariprazine, lurasidone, and brexpiprazole. Regarding the impact of aripiprazole on quality of life, 14 observational studies [49,58,74,83,84,85,91,98,99,100,101,102,103,104] and one randomized controlled trial [86] assessed the efficacy of the oral formulation of aripiprazole. Aripiprazole long-acting and aripiprazole lauroxile were included in six papers.

In the vast majority of studies including an oral antipsychotic, a positive effect on quality of life was found. Only the study by Fervaha et al. [105] found a modest effect of olanzapine, perphenazine, and quetiapine on quality of life and seven other studies [82,91,93,94,100,106,107] found no effect of oral antipsychotics on quality of life. Among second- and third-generation oral antipsychotics, aripiprazole and lurasidone were associated with the highest levels of improvement in quality of life (Table 1).

When patients were treated with clozapine, a significant improvement in QoL at all the assessments was found [52,90].

The majority of studies confirmed a positive effect of depot/long-acting injectable antipsychotics on quality of life, while only six studies [50,78,108,109,110,111] did not find any positive effect on quality of life (Table 2). In the study by Nasrallah et al. [77], aripiprazole lauroxil and paliperidone palmitate were found to be associated with a stable improvement in quality of life, and to also have a good safety and tolerability profile.

In four studies [112,113,114,115], patients were treated with a combination of oral and depot/LAI formulations. Only the study by Sugawara et al. [115] did not report details on the type of pharmacological agents used, and no effects were found (Table 3).

**Table 1:** Studies focusing oral antipsychotics and quality of life (N = 60).

	Author (Year), Country	Study Design	Setting	Sample (N, Diagnosis)	Diagnostic Assessment	Pharmacological Treatment	Outcome	Outcome Assessment	Results	Imp QoL
1.	Morss et al. (1993), USA [88]	Observational, cross-sectional	IN/OUT	33, SCH		Clozapine	To provide a quantitative measure of the impact of drug side-effects on patients’ QoL through a survey.	VAS, SG	VAS mean values for QoL with each side-effect: Akathisia > Tardive dyskinesia > Parkinsonism; SG mean values for QoL with each side-effect: Akathisia = Tardive dyskinesia > Parkinsonism.	y
2.	Awad et al. (1997), Canada [79]	Double-blind RCT		205, SCH	DSM-III-R, PANSS, CGI, extrapyramidal symptoms checklist, AIMS	Haloperidol vs. Remoxipride	To assess negative symptoms and QoL using smaller Haloperidol doses vs. Remoxipride.	Modified version of SIP	Comparable improvement in negative symptoms and in global and multidimensional self-assessment of QoL among the two groups. SIP alertness subfactor showed a statistically significant difference (Remoxipride without sedating properties).	y
3.	Hamilton et al. (1998), USA [80]	Double-blind RCT	IN	335	DSM-III-R diagnosis, with an acute exacerbation. Excluded if organic diseases, substances within 3 months, serious suicidal risk.	Olanzapine vs. Haloperidol	To assess the impact of treatment with Olanzapine compared with Haloperidol and placebo on improvements in clinical symptoms and QoL.	BPRS, CGI, SANS, QLS	Olanzapine significantly superior to haloperidol in reducing negative symptoms in acute treatment (SANS) and providing improvement in QoL (QLS).	y
4.	Bobes et al. (1998), Spain [116]	Observational, longitudinal	OUT	318 SCH	ICD-10	Risperidone	To assess the effect of Risperidone monotherapy on disability and QoL at baseline and months 2, 4, and 8.	BPRS, CGI, UKU, WHO-DDS, SF-36	WHO/DDS scores significantly decreased; SF-36 showed an improvement after 8 months with Risperidone treatment. Improvement in QoL in females, paranoid, and patients with history of use or abuse of substances.	y
5.	Galletly et al. (1997), Australia [89]	Observational, cross-sectional	OUT	19 SCH	DSM-III-R, SADS-L	Clozapine	To assess the correlations between reduction in symptoms, changes in neuropsychological test performance, and improvement in QoL.	PANSS, QLS, WAIS-R Digit Symbol Substitution, Block Design and Similarities, Controlled Oral Word Association, Category Instance Generation, Selected Reminding Test, Consonant Trigram, WISC-R, Mazes	Reduction in negative symptoms and general psychopathology associated with a better QoL.	y
6.	Meltzer et al. (1990), USA [73]	Observational, longitudinal	IN	38 SCH	DSM-III-R, SADS-L	Clozapine	To assess the effect of Clozapine on the QoL of patients during 6 months of treatment.	QLS, BPRS	At 6 months, besides a significant improvement in total BPRS, the total QLS score increased by 59.9% in the mean and 100% in the median score. The largest mean increase occurred for interpersonal role and intrapsychic foundations. At 12 months, rehospitalization rate was reduced by 83% independently of the 6-month QoL ratings.	y
7.	Dima et al. (2015), Romania [84]	Observational	IN/OUT	131 SCH, SPH, SAD, DD, BPD	DSM-IV-TR, PANSS	Haloperidol, Olanzapine, Risperidone, Quetiapine, Aripiprazole	To assess the evolution of patients’ reported quality of life when treated with antipsychotics, in naturalistic settings.	CGI, MOS SF-36, Q-LES-Q Short Form	Patients’ reported quality of life, satisfaction with treatment, and components of quality of life had a favorable evolution during the 12 months of follow-up. The differences among treatment groups were not statistically significant, with few exceptions.	y
8.	Li et al. (2015), China [95]	Observational, cross-sectional	IN/OUT	13013 SCH	DSM-IV, ICD-10	Clozapine	To explore the demographic and clinical correlates of Clozapine treatment and its independent associations with treatment satisfaction and QoL.	CGI-S, TESS, SF-12	Patients using Clozapine presented decreased satisfaction with treatment by the families, but similar QOL than patients not prescribed clozapine.	y
9.	Antunes de Araùjo et al. (2015), Brazil [117]	Observational, cross-sectional	OUT	108 SCH	ICD-10, DSM-IV	Olanzapine vs. Risperidone	To assess quality of life and side-effects among patients suffering from schizophrenia and assuming Olanzapine or Risperidone.	EQ-5D, UKU, SAS	The mean Quality-Adjusted Life-Year (QALY) value was higher for Risperidone than for Olanzapine users, who presented higher levels of asthenia, lassitude, fatigue, dystonia, and tremor.	y
10.	Hashimoto et al. (2015), Japan [118]	Observational, longitudinal		29 SCH	DSM-IV-TR, PANSS	Quetiapine	To assess long-term efficacy and tolerability of Quetiapine in patients with schizophrenia who switched from other antipsychotics because of inadequate therapeutic response at 3, 6, and 12 months.	BACS, CGI, GAF, JSQLS, AIS, DAI-30, DIEPSS	Statistically significant improvements were observed in all subscores of the PANSS, the GAF, and the symptoms and side-effects subscales of the JSQLS, the DIEPSS, the AIS, and the prolactine level.	y
11.	Montgomery et al. (2015), Australia [119]	Observational, longitudinal	OUT	475 SCH	DSM-IV, BPRS,	Olanzapine	To assess change in symptoms and quality of life among patients with schizophrenia who switched from typical antipsychotics to Olanzapine.	CGI-S, WHOQOL-BREF	Symptoms and health-related quality of life (HRQOL) both improved significantly over the 12 months of treatment.	y
12.	de Araújo et al. (2014), Brazil [92]	Observational, cross-sectional	OUT	218 SCH	DSM-IV, ICD-10	Olanzapine, Risperidone, Ziprasidone, Quetiapine, Clozapine	To assess the impact of atypical antipsychotic treatment on QoL and the adverse effects.	EQ-5D, UKU SERS, SAS	Besides significant differences in side-effects, EQ-5D scores showed that all drugs, except Olanzapine, significantly impacted mobility. An total of 63.6% of Clozapine users reported mobility problems; Clozapine and Ziprasidone users had difficulties with usual activities; Ziprasidone and Clozapine users experienced pain and/or discomfort; and 72.8% of Clozapine users reported anxiety and/or depression.	y
13.	Schreiner et al. (2014), Germany [104]	Non-interventional, naturalistic study	IN/OUT	4051 SCH	Not specified	Paliperidone ER, Amisulpride, Aripiprazole, Olanzapine, Quetiapine, Risperidone, Ziprasidone	To assess long-term outcomes related to initiation of Paliperidone ER and other oral antipsychotics in a naturalistic setting.	CGI-S, CGI-SCH, PSP, SF-12, VAS	Paliperidone ER was associated with greater improvements from baseline to endpoint in SF-12 physical scores.	y
14.	Awad et al. (2014), Canada [120]	Open-label trial	OUT	235 SCH, SAD	DSM-IV	Lurasidone	To assess health-related QoL changes among patients with schizophrenia who switched from their current antipsychotic to Lurasidone.	PETiT, SF-12	With regard to SF-12, improvements were observed for all patients, for those who switched from Quetiapine or Aripiprazole.	y
15.	Gattaz et al. (2014), Brazil [121]	Observational, longitudinal	OUT	213 SCH	DSM-IV-TR, PANSS	Risperidone vs. Paliperidone ER	To assess the impact of switching from oral Risperidone to flexibly dosed oral Paliperidone extended-release on schizophrenia symptoms, satisfaction, and quality of life.	PSP, CGI-S, SF-36, PSQI	Significant improvements from baseline in PANSS, personal and social functioning, and health-related quality of life (Short-Form 36), particularly on the Mental Component Summary.	y
16.	Gutiérrez Fraile et al. (2013), Spain [122]	Observational, longitudinal	IN/OUT	208 SCH, SAD, SPH	DSM-IV-TR, BPRS	Ziprasidone	To assess the long-term outcome of switching to Ziprasidone in terms of clinical efficacy, quality of life, functionality, and safety measures.	CGI-S, CGI-I, GAF, WHO-DAS-II, SF-12	Statistically significant improvements were observed in the GAF, WHO-DAS-II, and SF-12.	y
17.	Naber et al. (2013), Germany [123]	RCT	OUT	798 SCH, SAD, SPH	DSM-IV-TR, CGI-SCH, CDSS	Quetiapine XR vs. Risperidone	To assess the long-term subjective well-being of outpatients with schizophrenia, treated with either Quetiapine XR or oral Risperidone at a flexible dose in a naturalistic setting for a period of one year.	SWN-K, EQ-5D, UKU	Patient quality of life, measured using the EQ-5D health profile, was similar for both treatment groups at month 6 and month 12.	y
18.	Yeh et al. (2014), Taiwan [103]	Observational, longitudinal	OUT	23 SCH	DSM-IV-TR, BPRS	Aripiprazole	To assess neurocognitive effects of oral Aripiprazole on patients suffering from schizophrenia aged 12–26 at 4, 12, and 24 weeks of treatment.	CGI-S, WHOQOL, CPT, WCST	Statistically significant improvements in BPRS, CGI-S, and WHOQOL scores in certain (but not all) subcategories of cognitive measures including CPT detectability and total errors and perseverative errors on the WCST.	y
19.	Lin et al. (2013), Taiwan [124]	RCT	IN	96 SCH	DSM-IV, PANSS, CDSS, AIMS	Amisulpride + Sulpride vs. Amisulpride	To compare full-dose Amisulpride monotherapy and a combination of low-dose Amisulpride plus low-dose Sulpride in efficacy, safety, and quality of life for treatment of newly hospitalized schizophrenic patients with acute exacerbation.	CGI-S, GAF, SAS, BAS, SF-36	Similar results in terms of clinical characteristics at baseline, response rates, changes in all psychopathology measures, quality of life, and all side-effect scales after 6 weeks of treatment.	y
20.	Bervoets et al. (2012), Belgium [99]	Observational, longitudinal		361 SCH	DSM-IV-TR	Aripiprazole	To assess changes in verbal cognition and the predictive value of a cognitive improvement on quality of life.	Q-LES-Q, CGI-S, CVLT Verbal Fluency	The improvement in quality of life is explained by the effect of Aripiprazole on the CGI-S score, though the leisure and social relation scales of the Q-LES-Q also independently correlated with verbal fluency.	y
21.	Kusumi et al. (2012), Japan [125]	Randomized non-controlled trial	IN/OUT	118 SCH	DSM IV, PANSS	Olanzapine orally disintegrating tablet vs. oral standard tablet	To clarify whether or not body weight change differed between olanzapine ODT (orally disintegrating tablet) and OST (oral standard tablet) treatments in Olanzapine-naïve schizophrenia patients.	PANSS, GAF, WHO-QOL26, DAI, UKU	No significant difference was found between the two groups in any metabolic measure, efficacy, tolerability, WHO-QOL26, or DAI-10 score.	y
22.	Peuskens et al. (2012), Belgium [102]	Observational, longitudinal	IN/OUT	361 SCH	DSM-IV	Aripiprazole	To evaluate the effectiveness of 12-week treatment with Aripiprazole in schizophrenia.	CGI-S, CGI-TI, IAQ, PGI-I, Q-LES-Q, DAI-10, POM	Patients reported significantly improved quality of life and overall.	y
23.	Ye et al. (2011), Japan [126]	Observational, longitudinal	IN/OUT	1850 SCH	DSM IV, CGI-SCH	Olanzapine	To identify characteristics of patients with schizophrenia who continue Olanzapine therapy for 1 year.	EQ-5D-VAS	Continuers showed significantly greater early (3-month) improvement in global symptom severity. Logistic regression found that continuation was significantly predicted by longer illness duration, lower positive symptoms, higher negative symptoms, and better health-related quality of life.	y
24.	Ye et al. (2012), Japan [127]	Observational, longitudinal	IN/OUT	258 SCH	DSM IV, CGI-SCH, PANSS	Risperidone vs. Olanzapine	To assess clinical and functional outcomes following a switch from Risperidone to Olanzapine in 1 year.	EQ-5D-VAS	Patients experienced clinically and statistically significant improvements in health-related quality of life, and paid work rates.	y
25.	Liu-Seifert et al., (2012), USA [101]	Observational, longitudinal	IN/OUT	2193 SCH, SAD, SPH	DSM-IV-TR	Olanzapine, Risperidone, Quetiapine, Ziprasidone, Aripiprazole	To assess differential responses to treatment with various atypical antipsychotics in specific symptom domains and in quality of life.	PANSS, QLS	Significant improvement in QLS may best predict treatment adherence. Olanzapine-treated patients experienced significantly greater improvements in QLS than patients treated with the other atypical antipsychotics examined.	y
26.	Mahmoud et al. (2011), UK [128]	Observational, longitudinal	Not specified	42 SCH	DSM-IV	FGAs and (non-Clozapine) SGAs	To test if sexual disfunctions due to AP treatment may contribute to reduced quality of life.	ISF-SR, QLS	Change in sexual function was associated with change in quality of life.	y
27.	Hsieh et al. (2010), Taiwan [98]	Observational, longitudinal	Not specified	245 SCH, SAD	DSM-IV	Aripiprazole	To evaluate the overall long-term effectiveness of Aripiprazole.	CGI, BPRS, QOL	Compared to baseline scores, after 64 weeks of treatment showed significant improvements.	y
28.	Lin et al. (2010), Taiwan [81]	Observational, longitudinal	IN	88 SCH	SCID—DSM-IV	Risperidone + Haloperidol vs. Risperidone	To compare efficacy and safety of Risperidone monotherapy versus low-dose Risperidone plus low-dose Haloperidol.	CGI-S, PANSS, CDSS, GAF, AIMS, SAS, UKU	The two treatment groups are similar in efficacy, life quality, and other safety profiles.	y
29.	Aitchison et al. (2011), UK [58]	Observational, longitudinal	OUT	27 PD	Not specified	Aripiprazole	To compare costs and outcomes of the treatment regime before and after the introduction of Aripiprazole.	QLS, CSRI	Significant increase in the QLS between baseline and one year. Also, reductions over time in total direct and indirect cost.	y
30.	Kinon et al. (2010), USA [129]	RCT	IN/OUT	628 SCH, SAD	DSM-IV	Risperidone	To evaluate the effects of early response/non-response to an atypical antipsychotic across multiple outcome measures.	PANSS, MADRS, QLS, SOFI, SWN	Improvement across multiple outcome dimensions was not delayed, referring to improvement in psychiatric symptoms. Patients who showed an early response to antipsychotic also showed early and consistent improvement in functioning, quality of life, and subjective well-being.	y
31.	Kim and Kim (2009), South Korea [130]	Observational, cross-sectional	OUT	30 SCH	DSM-IV	Risperidone	To examine the association of adverse drug effects with subjective well-being in patients with schizophrenia receiving stable doses of Risperidone.	SWN, LUNSERS, DIEPSS	Adverse effects, particularly EPS and akathisia, are significantly associated with subjective well-being.	y
32.	Sacchetti et al. (2009), Italy [90]	RCT	Not specified	147 SCH	DSM-IV	Ziprasidone or Clozapine	To compare efficacy and safety of Ziprasidone and Clozapine in severely ill patients with schizophrenia and a history of resistance and/or intolerance to multiple cycles with antipsychotic medications.	PANSS, CGI, CGI-I, GAF, CDSS, DAI-10, SAS, BAS, AIMS	Both ziprasidone and clozapine have comparable efficacy coupled with satisfactory general safety and tolerability in schizophrenia patients with a history of multiple resistance and/or intolerance to antipsychotics.	y
33.	Popolo et al. (2010), Italy [49]	Observational, longitudinal	Not specified	15 SCH, SPH, SAD, DD, BPD	Not specified	Aripiprazole	Relationship between cognitive function, social functioning, and quality of life in patients with FEP.	WCST, SEL/AT, FAS, CPM, BPRS, CGI, HoNOS, GAF, Q-LES-Q	Social functioning and quality of life are related, but independent of cognitive impairment.	y
34.	Ishigooka et al. (2021), Japan [74]	Open-label, three-arm, randomized, parallel-group study	OUT	251 SCH	DSM-IV-TR	Aripiprazole, Blonanserin, Paliperidone	52-week discontinuation rate, remission rate, symptom alleviation, aggravation and recurrence, social functioning, and quality of life.		QoL improvement at start of monotherapy, and 26 and 52 weeks in the overall cohort.	y
35.	Iyo et al. (2021), Japan [75]	RCT	OUT	289 SCH	DSM-IV-TR	Lurasidone	Treatment-emergent adverse events; emergence of suicidality; symptom severity reduction; quality of life; treatment discontinuation.	CGI-S, PANSS, EQ-5D-3L	Increase in QoL measures: at week 12, overall mean increase of 0.097 ± 0.190 and 0.028 ± 0.141 relative to double-blind and open-label baseline on EQ-5D-3L index scores, respectively, and overall mean increase in EQ VAS scores of 16.8 ± 24.1 and 5.3 ± 18.8.	y
36.	Verma et al. (2020), India [96]	Observational, longitudinal	IN/OUT	52 SCH	DSM-IV	Clozapine	Improvement in psychopathology, functioning, QoL, side-effects (at 3 months).	WHOQL-BREF	Significant improvement in all domains of the two scales (all *p* values < 0.001 except for WHOQOL-Bref social relationships subscale, *p* = 0.002).	y
37.	Veselinovic et al. (2019), multi-site [86]	RCT	IN/OUT	114 SCH	ICD-10	Haloperidol, Flupentixol; Aripiprazole, Olanzapine, Quetiapine	Cognitive performance, psychopathology, clinical functioning, QoL (at 6 and 24 weeks).	SF-36	At 24 weeks, higher QoL at SF-36 in the SGA group (FGA: 83.8 ± 17.6; SGA: 97.9 ± 11.0; *p* = 0.04, ES 0.42).	y
38.	Sahni et al. (2016), India [52]	RCT	IN/OUT	63 FEP	ICD-10	Clozapine vs. Risperidone	Symptom severity, QoL, side-effects.	PANSS, WHOWOL-BREF, ASEX, GASS	Significant improvement in QoL in the clozapine subgroup at all the assessments, with significant between-group difference.	y
39.	Lee et al. (2016), China [53]	Observational, cross-sectional	IN/OUT	285 FEP	DSM-IV	Haloperidol, Olanzapine, Risperidone, Amisulpride, Sulpride, Quetiapine	QoL, side-effects, functioning.	SF-12	Scores at the SF-12 were significantly higher in patients taking amisulpride than in those prescribed with the other drugs.	y
40.	Grunder et al. (2016), multi-site [85]	RCT	IN/OUT	136 SCH	ICD-10	Haloperidol, Flupentixol; Aripiprazole, Olanzapine, Quetiapine	QoL, subjective well-being, symptom severity, side-effects.	SF-36	Mean AUC values for the SF-36 were significantly higher in the SGA than in the FGA group.	y
41.	Awad et al. (2016), multi-site [131]	Observational, longitudinal	OUT	144 SCH, SAD	DSM-IV-TR	Lurasidone	Quality of life.	PETit, SF-12	Mean PETiT total score significantly improved from 34.9 ± 9.3 at baseline to 39.5 ± 8.9 at extension baseline and 39.1 ± 9.0 at extension endpoint, representing improvements of 4.5 ± 7.9 and 5.1 ± 7.2 points, respectively (*p* < 0.001).	y
42.	Kao et al. (2011), Taiwan [83]	Observational, cross-sectional	IN	104 SCH, SAD	DSM-IV	Haloperidol, Risperidone, Quetiapine, Amisulpride, Aripiprazole, Ziprasidone, Zotepine, Olanzapine, Clozapine	Reliability and validity of the Taiwanese version of the WHOQOL-BREF assessment. Secondly, association of psychosocial characteristics, severity of symptoms, insight measures, and side-effects of antipsychotics by using subjective QOL.	PANSS, WHOQOL-BREF, ESRS, BDI, ACL, BHS, SSI	As predicted, age, onset of illness, insight measures, symptom severity, general psychopathology, and antipsychotic-induced side-effects were all significantly related to the QOL scores.	Y
43.	Hasan et al. (2019), multi-site [132]	Observational, cross-sectional	OUT	157 SCH	DSM-5	FGAs and SGAs	Association between QoL and sociodemographic/clinical variables.		QoL was positively correlated with receiving an SGA (r = 0.38) and negatively correlated with medication side-effects (DIEPSS score) (r = −0.53). The latter was significantly associated with QoL at the regression analysis (PSW and SLE subdomains: β = 0.48, β = −0.34, *p* < 0.05).	y
44.	Mauri et al. (2015), Italy [133]	RCT	OUT	133 SCH	DSM-IV, PANSS	Paliperidone ER	To assess efficacy, safety, and patients’ perception of their social functioning and well-being when risperidone ER is taken.	CGI-s, PSP, DAI-30, SWN−20,	Significant reduction in the total PANSS score. The mean CGI-S scores significantly decreased. A mean improvement in PSP scores from baseline was statistically significant at week 6 and at endpoint. Patients’ attitudes to treatment (mean DAI-30 scores) improved significantly.	No qol
45.	Hou et al. (2015b), China [94]	Observational, cross-sectional	OUT	623 SCH	ICD-10, BPRS	Clozapine	To assess the demographic and clinical correlates of clozapine treatment in relation to quality of life.	SAS, SF-12, ITAQ	No significant differences between the patients with and without clozapine in QoL domains.	no
46.	Shrivastava et al. (2012), Canada [91]	Observational, cross-sectional	Not specified	116 SCH	DSM-IV, PANSS, HAM-D	Clozapine, Risperidone, Olanzapine, Quetiapine, Aripiprazole, Ziprasidone	To assess patterns of antipsychotic usage in patients with longstanding psychosis and their relationship to social outcomes.	CGI-S, GAF, WHOQOL-BREF	No significant differences in recovery on CGI, QOL, or GAF between groups of antipsychotic drugs.	no
47.	Stahl et al. (2010), USA [82]	RCT	OUT	599 SCH, SAD	DSM-III-R	Ziprasidone vs. Haloperidol	To compare the negative symptom efficacy and treatment outcomes of Ziprasidone versus Haloperidol.	PANSS, QLS, SARS, BAS, MDB	No significant differences for Ziprasidone versus Haloperidol.	no
48.	Lin et al. (2017), Taiwan [106]	RCT	IN	90 SCH	DSM-IV	Olanzapine 10 mg vs. Olanzapine 5 mg plus Trifluoperazine 5 mg	Symptom severity, functioning, side-effects, QoL.	SF-36	No significant differences at the SF-36 domains between the two subgroups.	no
49.	Kim et al. (2014), South Korea [93]	Observational, longitudinal, and cross-sectional		40 SCH	DSM-IV, SCID-IV, PANSS, Beck Depression Inventory (BDI)	Clozapine	To assess subjective well-being, schizophrenia symptoms, and depressive symptoms before and 8 weeks after the initiation of treatment with Clozapine.	CGI-S, SWN	Before and after Clozapine administration, the subjective well-being score had significant negative correlations with the PANSS depression factor score and the BDI score.	no
50.	Huang et al. (2013), Taiwan [100]	Observational, longitudinal	OUT	42 SCH	DSM IV, BPRS	Aripiprazole	To determine the clinical outcomes of Aripiprazole treatment in adolescents and young adults with schizophrenia spectrum disorders.	MINI, CGI-S, BPRS, WHOQOL-BREF	Psychotic symptoms, but not quality of life, globally improved from baseline scores by the endpoint of the study.	no
51.	Melo Chaves et al. (2013), Brazil [107]	Observational, cross-sectional	OUT	115 SCH	ICD-10, DSM IV	Olanzapine vs. Risperidone	To compare the effects of treatment with an atypical antipsychotic drug (olanzapine or risperidone) on quality of life.	UKU, QLS-BR	QoL was impaired in patients using olanzapine and in those using risperidone.	no
52.	Fervaha et al. (2014), Canada [105]	RCT		753 SCH	DSM-IV, SCID-I, PANSS, CDSS,	Olanzapine, Perphenazine, Quetiapine, Risperidone or Ziprasidone	To assess in a perspective of 12 months the effects of antipsychotic medication on overall life satisfaction in patients with chronic schizophrenia.	CGI-S, QLS, ITAQ, Lehman Quality of Life Interview, DAI, EPS Score	Modest improvements in overall life satisfaction with no differences between antipsychotic treatments.	mod
53.	Ye et al. (2014), USA [134]	Observational, longitudinal	OUT	330 SCH	DSM-IV or ICD-10	Olanzapine	To assess predictors of early treatment response to Olanzapine clinical and functional outcomes for early responders compared with early non-responders.	CGI-S	Early responders were significantly more likely to meet treatment response criteria at endpoint and had significantly greater improvement in symptoms and functional outcomes.	
54.	Kilian et al. (2012), Germany [135]	Observational, longitudinal	OUT	374 SCH, SAD	ICD-10	Quetiapine vs. Olanzapine or Risperidone	To examine the effects of Quetiapine in comparison with Olanzapine and Risperidone on clinical outcomes and quality of life in patients with schizophrenia and schizoaffective disorder in routine care.	PANSS, GAF, SAS, QOL, LQoLP, MARS	Quetiapine and Risperidone are less effective in preventing the need for psychiatric inpatient care than Olanzapine.	
55.	Nilsen et al. (2012), Denmark [97]	RCT	IN/OUT	50 SCH	ICD-10, PANSS	Clozapine + Sertindole vs. Clozapine + placebo	To assess Sertindole augmentation in Clozapine treatment on clinical outcomes.	PANSS, CGI, UKU, QoL-BREF	Clozapine augmentation with Sertindole was not superior to placebo.	no
56.	Adrianzen et al. (2010), Perù [48]	Observational, longitudinal	OUT	16091 SCH	ICD-10 or DSM-IV	FGAs and SGAs	To explore the relative association of adverse events with health-related quality of life (HRQL) in patients suffering from schizophrenia treated with antipsychotics.	EuroQoL-VAS	Association between each adverse event and HRQL.	no
57.	Gaebel et al. (2011), Germany [136]	RCT	IN	44 SCH	ICD-10	FGAs and SGAs	To compare the relapse preventive efficacy of maintenance treatment or targeted intermitted treatment in FEP patients.	PANSS, CGI, GAF, SANS, HAM-D, CDSS, EPS, HAS, UKU, DAI, LQLP, SWN	Maintenance treatment is more effective than targeted intermitted treatment in preventing relapses.	
58.	Li et al. (2010), Taiwan [137]	Observational, cross-sectional	OUT	90 SCH	DSM-IV	FGAs and SGAs	To assess symptom resolution rates and associated factors among medicated and clinically stable Chinese schizophrenia patients.	PANSS, UKU, SAS, GAF, SWN	Consistent with studies of Caucasian patients, one third of clinically stable Chinese patients met the resolution criteria, as well as having fewer general side-effects and better global functioning and subjective well-being.	
59.	Roberts et al. (2010), USA [138]	Observational, longitudinal	OUT	223 SCH, SAD	DSM-IV	Olanzapine vs. Quetiapine	To evaluate whether individuals treated with Olanzapine or Quetiapine achieved improvements in social cognition.	SCRT	Participants in both medication groups significantly but modestly improved on three out of four social cognition subscales.	Y
60.	Lambert et al. (2010), Germany [56]	Controlled clinical trial	IN/OUT	120 SCH	SCID-I, DSM-IV	Quetiapine IR	To evaluate the effectiveness of intensive Assertive Community Treatment with quetiapine IR.	PANSS, CGI-S, GAF, MVSI, MLCI, Q-LES-Q-18, SWN-K, SWAM, SES, CSQ-8	Compared to standard care intensive, Assertive Community Treatment as part of integrated care could improve 1-year outcome.	

IN: inpatients; OUT: outpatients. SCH: schizophrenia; SPH: schizophreniform disorder; SAD: schizoaffective disorder; DD: delusional disorder; BPD: brief psychotic disorder; FEP: first-episode psychosis; PD: psychotic disorder. QoL: Quality of Life. RCT: randomized controlled trial. FGA: First-Generation Antipsychotic; SGA: Second-Generation Antipsychotic. Imp QoL: Improvement in quality of life. Diagnostic and Outcome Assessment tools: ACL: Anxiety Checklist; AIMS: Abnormal Involuntary Movement Scale; AIS: Acceptance of Illness Scale; ASEX: Arizona Sexual Experience Rating Scale; BACS: Brief Assessment Cognition Schizophrenia; BAS: Barnes Akathisia Scale; BDI: Beck Depression Inventory; BHS: Beck Hopelessness Scale; BPRS: Brief Psychiatric Rating Scale; CDSS: Calgary Depression Scale for Schizophrenia; CGI: Clinical Global Impression; CPM: Colored Progressive Matrices; CPT: Continuous Performance Test; CSQ-8: Client Satisfaction Questionnaire; CSRI: Client Service Receipt Inventory; CVLT: California Verbal Learning Test; DAI: Drug Attitude Inventory; DIEPSS: Drug-induced Extrapyramidal Symptoms Scale; DSM: Diagnostic and Statistical Manual of Mental Disorders; ESRS: Extrapyramidal Symptoms Rating Scale; FAS: Verbal Fluency Task; GAF: Global Assessment of Functioning; GASS: Glasgow Antipsychotic Side-Effect Scale; HAM-D: Hamilton Rating Scale for Depression; HoNOS: Health of the Nation Outcome Scale; IAQ: Investigator’s Assessment Questionnaire; ICD-10: International Classification of Diseases 10th Revision; ISF-SR: Derogatis Interview for Sexual Functioning; ITAQ: Insight and Treatment Attitudes Questionnaire; JSQLS: Japanese version of the Schizophrenia Quality-of-Life Scale; LqoLP: Lancashire Quality-of-Life Profile; LUNSERS: Liverpool University Neuroleptic Side-Effect Rating Scale; MADRS: Montgomery–Åsberg Depression Rating Scale; MARS: Medication Adherence Rating Scale; MDB: Movement Disorder Burden scale; MINI: Mini-International Neuropsychiatric Interview; MLCI: Modified Location Code Index; MVSI: Modified Vocational Status Index; PANSS: Positive and Negative Syndrome Scale; PETiT: Personal Evaluation of Transitions in Treatment; PGI-I: Patient Global Impression-Improvement; POM: Preference of Medicine Questionnaire; PSP: Personal and Social Performance scale; PSQI: Pittsburgh Sleep Quality Index; Q-LES-Q: Quality-of-Life Enjoyment and Satisfaction Questionnaire; QLS: Quality-of-Life Scale; SADS: Schedule for Affective Disorders and Schizophrenia; SANS: Scale for the Assessment of Negative Symptoms; SARS: Staden Schizophrenia Anxiety Scale; SAS: Simpson Angus Scale; SCID: Structural Clinical Interview; SCRT: Social Cue Recognition Test; SEL/AT: Span Selective Attention; SERS: Side-Effect Rating Scale; SES: Socio-Economic Status; SF: Short-Form Health Survey; SG: Standard gamble; SIP: Sickness Impact Profile; SOFI: Schizophrenia Objective Functioning Instrument; SSI: Scale for Suicide Ideation; SWAM: Satisfaction with Antipsychotic Medication; SWN: Subjective Well-Being under Neuroleptic scale; TESS: Treatment Emergent Symptom Scale; UKU: UKU Side-Effect Rating Scale; VAS: visual analog scale; WAIS: Wechsler Adult Intelligence Scale; WCST: Wisconsin Card Sorting Test; WHO-DAS: World Health Organization-Disability Assessment Schedule; WHO-DDS: World Health Organization Disability Diagnostic Scale; WHOQOL: World Health Organization Quality-of-Life assessment; WISC: Wechsler Intelligence Scale for Children.

**Table 2:** Studies focused on long-acting antipsychotics and quality of life (N = 33).

	Author (Year), Country	Study Design	Setting	Sample (N, Diagnosis)	Diagnostic Assessment	Pharmacological Treatment	Outcome	Outcome Assessment	Results	Imp Qol
1.	Larsen and Gerlach (1996), Denmark [87]	Observational, cross-sectional	OUT	53 SCH	ICD-10	Ris(z)flupentixol decanoate, Zuclopentixol decanoate, Perfenazine decanoate	Attitude of patients to maintain depot therapy, side-effects, mental state, and quality of life.	PANSS, 14-item questionnaire evaluating patients’ attitude to treatment, PGWS, QLS, UKU	The PGWS score is relatively high. No correlation between patients’ VAS rating of their QoL or PGWS and age, duration of illness, side-effects, UKU, and PANSS scores.	y
2.	Niolu et al. (2015), Italy [139]	Observational, longitudinal	OUT	27 SCH	DSM-IV-TR	Risperidone LAI	Adherence to treatment, quality of life, and subjective well-being in non-adherent patients with schizophrenia.	SAPS, SANS, SWN, QLS	Increase in monthly mean values of SWN (from the eighth month) and QLS (from the eighteenth month) correlated with reduction in SAPS and SANS.	y
3.	Cervone et al. (2015), Italy [47]	Observational, cross-sectional	OUT	7 FEP	DSM-IV-TR	Paliperidone palmitate (N = 6), Olanzapine pamoate (N = 1)	Efficacy of long-acting antipsychotics in patients presenting FEP.	BPRS, HoNOS, GAF, ESRS	Overall improvement in terms of reduced psychotic symptoms, improved quality of life, and absence of extrapyramidal side-effects.	y
4.	Naber et al. (2015), Germany [140]	Rater-blinded RCT	OUT	295 SCH	DSM-IV-TR	Aripiprazole LAI vs. paliperidone palmitate	Comparing Aripiprazole LAI with Paliperidone palmitate in clinically stable patients.	QLS, CGI-S, IAQ	On QLS total score, a non-inferiority and established superiority of Aripiprazole LAI vs. Paliperidone palmitate were observed from baseline to week 28.	y
5.	Pietrini et al. (2015), Italy [141]	Longitudinal	OUT	26 SCH, SAD	DSM-5	Olanzapine LAI, Paliperidone LAI	The effects of switching from oral to the equivalent long-acting antipsychotic treatment in terms of subjective experience and quality of life at baseline and after 6 months in clinically stable patients.	PANSS, MADRS, YMRS, SWN-K, DAI-10, SF-36	A significant improvement in the attitude toward drug and subjective experience of treatment was observed. Initial non-remitters reported significantly higher health-related QoL and better functioning in all areas of daily living; initial remitters reported a significant improvement in general health, vitality, social functioning, and high perception of change in terms of health status.	y
6.	Rouillon et al. (2013), France [142]	Active-control RCT	IN/OUT	666 SCH, SAD	DSM-IV	Risperidone LAI vs. Quetiapine	Effectiveness of Risperidone LAI in comparison with oral Quetiapine in terms of functional recovery.	PANSS, CGI-S, SOFAS, SF-12, SQLS-R4	Significant improvements in SOFAS, SF-12, and SQLS-R4 scores were observed from baseline to month 24 with both in LAI Risperidonde and quetiapine users.	y
7.	Ascher-Svanum et al. (2014), USA [143]	RCT	OUT	524 SCH	DSM-IV, DSM-IV-TR	Olanzapine LAI vs. oral Olanzapine	Changes in functioning among patients suffering from schizophrenia (not hospitalized in the previous 8 weeks and at risk for relapse) with Olanzapine LAI treatment compared to oral Olanzapine.	PANSS, CGI-S, QLS	Both treatments led to an improvement in level of functioning: no significant differences between olanzapine-LAI and oral olanzapine were observed.	y
8.	Ascher-Svanum et al. (2011), USA [144]	RCT	IN	233 SCH	DSM-IV, DSM-TR	Olanzapine LAI	To assess whether early response predicted later response when using a long-acting injection (LAI) antipsychotic.	BPRS, PANSS, SF-36, QLS	Early responders had significantly greater improvement than early non-responders in QLS scores.	y
9.	Osborne et al. (2012), Australia [145]	Observational, cross-sectional	OUT	124 SCH		LAI Antipsychotics	To assess differences in health-related quality of life (HRQoL) for antipsychotic LAIs once every 2 weeks, 4 weeks, or 3 months.	HRQoL	An approximately 0.05 HRQoL difference exists between treatment options, with the highest related to 3-monthly injections.	y
10.	Peuskens et al. (2012), Belgium [146]	Observational, longitudinal	IN/OUT	1182 SCH		Olanzapine LAI	Treatment outcomes of patients with schizophrenia receiving maintenance treatment with Olanzapine LAI.	PANSS, QLS, CGI-S	The majority of all patients starting Olanzapine LAI treatment maintained or improved their symptom and functioning levels on Olanzapine LAI maintenance treatment.	y
11.	Witte el al. (2012), USA [147]	RCT	IN/OUT	404 SCH	DSM-IV, DSM-IV-TR	Olanzapine LAI	The effects of Olanzapine LAI on levels of functioning in acutely ill patients with schizophrenia.	BPRS, PANSS, QLS, SF-36	All three Olanzapine LAI treatment groups and the combined Olanzapine LAI group were superior to placebo on the QLS total score.	y
12.	Nasrallah et al. (2021), USA [77]	RCT	IN/OUT	200 SCH	DSM-5	Aripiprazole lauroxil vs. Paliperidone palmitate	Symptom severity reduction, caregiver burden, patients’ satisfaction with medication, and quality of life.		QoL stable across assessments for both medications, with values between good and fair.	Y
13.	Strunoiu et al. (2021), Romania [148]	Non-Randomized CT	IN	135 SCH	DSM-IV	Atypical antipsychotics LAI	Adverse effects, adherence to treatment and number of hospitalizations.	PANSS, WHOQOL-BREF	Significant improvement in QoL (higher WHOQOL-BREF score: median value 82 increasing to 94) at follow-up. Significant differences in all the WHOQOL-BREF domains.	Y
14.	Pietrini et al. (2021), Italy [149]	Observational, Longitudinal	OUT	35 SCH	DSM-IV-TR, DSM-5	Atypical generation antipsychotics, LAI formulation	Patient- and caregiver-reported perceived disability, subjective treatment, experience, and quality of life.	HRQoL, SF-36	Significant improvement in SF-36 scores at all subscales except for physical functioning and emotional role, with significant improvement at T2 and T1 compared to T0 and no significant variation in the second year; bodily pain improvement was not stable at T2, social functioning improved only at T2.	Y
15.	McEvoy et al. (2021), USA [76]	Post hoc analysis of two phase 3, multicenter, open-label safety studies	OUT	291 SCH	DSM-5	Aripiprazole lauroxil	Quality of life (changes from baseline to 124 weeks).	HRQoL	Significant improvement in mental HRQoL at all follow-up points, significant improvement in physical HRQoL at 112 weeks follow-up.	Y
16.	Giordano et al. (2020), Italy [54]	Observational, cross-sectional	IN	50 FEP	DSM-5	Aripiprazole LAI	Treatment efficacy on symptoms, safety, tolerability, and quality of life.	PANSS, CGI-S, WHOQOL-BREF, PSP, SF-36	Significant increase in SF-36, WHOQOL-BREF, and PSP scores over time; within-subject effect of time on every subscale. Significant main effects of age-at-onset on the environment and psychological WHOQOL domains, on the emotional, functioning, and general health perceptions SF-36 subscales, and on the personal, social relationships, and self-care PSP subscales.	Y
17.	Phahladira et al. (2020), South Africa [55]	Observational, longitudinal	IN/OUT	98 FEP	DSM-IV-TR	Flupenthixol decanoate	Psychopathology, functioning, quality of life (24 months follow-up) in FEP patients who were not treated with antipsychotics for >4 weeks and were never prescribed LAI.	QoL, SOFAS	Significant improvement in patient-rated QoL at month 12. No significant improvement after month 12. SOFAS scores were positively correlated with patient-rated overall QOL scores.	Y
18.	Llorca et al. (2018), multi-site [150]	Observational, longitudinal	IN/OUT	572 SCH	ICD-10	FGA and SGA LAI formulations	Symptom severity, functioning, insight, QoL, well-being, side-effects, and attitude toward medication.		Patients initiating SGA-LAI had better quality-of-life scores than those initiating FGA-LAI. Lowest QoL in subjects initiating FAI-LAI (incident LAI users).	y
19.	Naber et al. (2017), multi-site [151]	RCT	OUT	88 SCH	DSM-IV-TR	Aripiprazole LAI	Safety, quality of life, symptom severity up to 24 weeks.	QLS	Sustained improvements in QoL at follow-up. At week 24, the LSM change in QLS total score was 2.3. The aggregated LSM change from the baseline of the lead-in study to week 24 of the extension study was 11.5.	y
20.	Potkin et al. (2017), multi-site [152]	RCT	OUT	268 SCH	DSM-IV-TR	Aripipraole LAI vs. Paliperidone palmitate	Quality of life, symptom severity, safety, tolerability.	QLS, SWN-S, TooL	QLS improved significantly more with AOM 400 than with PP (*p* < 0.05 for both comparisons). At SWN-S and TooL, no significant among-treatment differences in improvement, but greater changes in the scores were evidenced for AOM.	y
21.	Sağlam Aykut (2019), Turkey [153]	CT	OUT	84 SCH	DSM-IV	Paliperidone palmitate	Symptom severity, side-effects, quality of life, medication adherence, and insight in clinically stable patients with schizophrenia treated for at least 6 months with paliperidone palmitate.	SCID-IV, PANSS, ESRS, UKU, SF-36	General health perception subscale was significantly higher in the paliperidone palmitate subgroup. No significant differences were observed in the other subscales.	
22.	Schreiner et al. (2015), Germany [108]	Rater-blinded RCT		775 SCH	DSM-IV	Paliperidone palmitate vs. oral antipsychotic monotherapy	Efficacy of paliperidone palmitate vs. oral antipsychotics for relapse prevention in patients experiencing an acute episode of schizophrenia and history of ≥2 relapses requiring psychiatric hospitalization in the preceding 24 months	PANSS, CGI-S, CGI-C, PSP, SF-36, EQ-5D, SWN-S, TSQM	Significantly greater improvement in EQ-5D score at month 12 in patients treated with oral antipsychotics.	
23.	Chiliza et al. (2015), South Africa [50]	Observational, longitudinal	IN/OUT	126 FEP	DSM-IV	Flupenthixol decanoate	Rate of non-response to first-line treatment in first-episode schizophrenia, symptom non-response, and demographic, baseline clinical, and early treatment response predictors of non-response.	SCID-IV, PANSS, CDS-S, Birchwood Insight Scale, Premorbid Adjustment Scale, SOFAS, NES, ESRS, WHOQOL-BREF, MATRICS MCCB	Patients with FEP, who did not respond in terms of symptoms reduction, present significantly worse conditions in social and occupational functioning, quality of life, and cognitive performance and had significantly higher NES scores.	n
24.	Lee et al. (2014), South Korea [109]	Observational, longitudinal	IN/OUT	472 SCH, SAD, SPH	DSM-IV	Risperidone LAI	Clinical and QoL outcomes in patients with schizophrenia or schizoaffective disorder treated with Risperidone LAI for 48 weeks.	PANSS, CGI-S, SQLS, SAS	Total scores of eight items of PANSS, CGI-S, SQLS, and SAS significantly reduced from baseline to endpoint in both intention-to-treat per-protocol (who completed the study) populations.	n
25.	Leatherman et al. (2014), USA [110]	RCT	OUT	369 SCH, SAD	DSM-IV	Risperidone LAI vs. oral antipsychotics	The risk of psychiatric rehospitalization and, secondly, symptoms, quality of life, and global functioning up to 24 months of follow-up.	SCID-IV, PANSS, QLS	No significant differences in treatment in 10 of 12 subgroups on psychiatric symptoms, quality of life, or time to hospitalization.	n
26.	Rosenhek et al. (2011), USA [111]	RCT	IN/OUT	369 SCH, SAD	DSM-IV	Risperidone LAI	Hospitalization, symptoms, quality of life, and functioning.	SCID-IV, CGI, DAI, PANSS, BSI, QLS, PSP, Quality of Well-Being scale, AIMS	Quality of life was not significantly improved with long-acting injectable risperidone as compared with control treatments.	n
27.	Di Lorenzo (2022), Italy [78]	Observational, cohort study	OUT	90 SCH	ICD-9	Haloperidol decanoate, paliperidone palmitate (1 month), paliperidone palmitate (3 months)	Urgent psychiatric consultations (number); psychiatric hospitalizations (number, days); adverse effects and BMI change; drop-outs and reasons; quality of life, functioning, clinical severity.	WHOQOL-BREF	No significant differences in WHOQOL-BREF total and subscale scores at 6 and 12 months between treatment groups. Negative association of WHOQOL-BREF score with medical comorbidity, socio-economic problems, length of inpatient stay during LAI treatment.	n
28.	Tsang et al. (2010), Hong-Kong [57]	Observational, cross-sectional	OUT	153 PD	DSM-IV	Conventional depot antipsychotic (CDA) and atypical depot antipsychotic (ADA)	Satisfaction level of psychiatrists and psychotic patients toward CDA and ADA on symptom management, role functioning, and side-effects.	2 questionnaires from the perspectives of psychiatrists and patients	Both groups shared similar attitudes toward clinical effectiveness and treatment efficacy of ADA and CDA. More patients were ambivalent toward relapse prevention of CDA than psychiatrists and three quarters of psychiatrists believed that ADA are associated with fewer side-effects. More than half of the patients showed negative attitudes toward the effectiveness of CDA on improving quality of life, work, and recreation. Psychiatrists were more aware about the limitation of CDA and severity of side-effects of CDA.	
29.	Chiliza et al. (2016), South Africa [51]	Longitudinal	IN/OUT	207 FEP	DSM-IV	Flupenthixol decanoate	Feasibility and effectiveness Flupenthixol decanoate in combination with an assertive monitoring program in FEO.	SCID-IV, PANSS, CDSS, CGI, SOFAS, WHOQOL-BREF	High response and remission rates, with significant improvements in social and occupational functioning and quality of life.	y
30.	Schmauss et al. (2010), Germany [154]	Non-randomized clinical trial	IN/OUT	253 SCH, SAD	ICD-10, PANSS (50–80)	Risperidone LAI	Effects of Risperidone LAI in patients following direct transition from oral risperidone compared with transition from other oral second-generation antipsychotics	PANSS, CGI-S, CGI-C, SWN-K, SF-12	Compared to risperidone pre-treatment, clinically stable patients with schizophrenia who are pre-treated with OQAZ (Olanzapine, Quetiapine, Amisulpride, Ziprasidone) might draw a stronger clinical benefit from direct transition to Risperidone LAI.	
31.	Lambert et al. (2010), Germany [155]	Observational, longitudinal		529 PD	DSM-IV	Risperidone LAI	Symptomatic and functional remission.	PANSS, GAF, SF-36	One in three patients with stable schizophrenia switching to Risperidone LAI experienced symptomatic remission, with combined symptomatic, functional, and quality-of-life remission in one in five patients.	
32.	Pietrini et al. (2018), Italy [156]	Observational, longitudinal	OUT	43 SCH	DSM-IV-TR, DSM-5	Aripiprazole, Olanzapine, Paliperidone LAI	Attitude toward medication, subjective experience of treatment, quality of life.	SF-36	Significant improvement in all SF-36 domains between T0 and T1 and between T0 and T2: general health, vitality, emotional role, mental health, physical functioning, physical role, bodily pain, and perceived social functioning.	y
33.	Isitt et al. (2016), USA [157]	RCT	IN/OUT	337 SCH	DSM-IV-TR	Risperidone LAI	Symptom severity, HRQoL, well-being, satisfaction with medications.	EuroQoL (EQ-5D-5L) VAS, SWN-S	Significant increase at the EQ-5D-5L VAS in the RBP-7000 120 mg group compared to placebo (*p* = 0.0212).	y

IN: inpatients; OUT: outpatients. SCH: schizophrenia; SPH: schizophreniform disorder; SAD: schizoaffective disorder; DD: delusional disorder; BPD: brief psychotic disorder; FEP: First-Episode Psychosis; PD: Psychotic Disorder. QoL: Quality of Life. RCT: randomized controlled trial. Imp QoL: Improvement in quality of life. Diagnostic and Outcome Assessment tools: AIMS: Abnormal Involuntary Movement Scale; ASI: Anxiety Sensitivity Index; BPRS: Brief Psychiatric Rating Scale; BSI: Brief Symptom Inventory; CDSS: Calgary Depression Scale for Schizophrenia; ESRS: Extrapyramidal Symptoms Rating Scale; CGI: Clinical Global Impression; DAI: Drug Attitude Inventory; HoNOS: Health of the Nation Outcome Scales; HRQoL: Health-related Quality of Life; IAQ: Investigator’s Assessment Questionnaire; MADRS: Montgomery–Åsberg Depression Rating Scale; MCCB: MATRICS Consensus Cognitive Battery; NES: Neurological Evaluation Scale; PANSS: Positive and Negative Syndrome Scale; PGWS: Psychological General Well-Being Schedule; PSP: Personal and Social Performance scale; QLS: Quality-of-Life Scale; SANS: Scale for Assessment of Negative Symptoms; SAPS: Scale for the Assessment of Positive Symptoms; SAS: Simpson Angus Scale; SCID: Structural Clinical Interview; SF: Short-Form Health Survey; SOFAS; Social and Occupational Functioning Assessment Scale; SQLS: Schizophrenia Quality-of-Life Scale; SWN: Subjective Well-Being under Neuroleptic scale; TooL: Tolerability and Quality-of-Life questionnaire; TSQM: Treatment Satisfaction Questionnaire for Medication; UKU: UKU Side-Effect Rating Scale; WHOQOL: World Health Organization Quality-of-Life assessment; YMRS: Young Mania Rating Scale.

**Table 3:** Non-specified antipsychotic therapies (N = 11).

	Author (Year), Country	Study Design	Setting	Sample (N, Diagnosis)	Diagnostic Assessment	Pharmacological Treatment	Outcome	Outcome Assessment	Results	Imp QoL
1.	Browne et al. (1998), Ireland [35]	Observational, cross-sectional	OUT	42 SCH	DSM-III-R		Relationship between QoL, insight, and subjective response to neuroleptics.	QLS, IS, DAI	No significant relationship between QoL and level of insight. A dysphoric response to neuroleptics influences social and interpersonal functioning, sense of psychological well-being, and participation in daily activities.	n
2.	Awad et al. (1997b), Canada [34]	Observational, cross-sectional	OUT	62 SCH	DSM-III-R		Symptom severity, side-effects, subjective responses, psychosocial functioning, and self-rated global QoL.	PANSS, AIMS, HAS, SPS, DAI, GAF, Gurin’s Global QOL question	Moderate impairment of functioning (especially in employment, intimate relationships, and child care). Self-rated QoL significantly correlated with clinical symptoms, akathisia, and subjective responses to antipsychotic drug, but not with abnormal movements or psychosocial functioning.	
3.	Voruganti et al. (1998), Canada [36]	Observational, longitudinal	OUT	63 SCH	DSM-IV		Self-reported QoL at weekly intervals over a period of 4 weeks and to examine the effect of illness and treatment of QoL appraisal in stable patients.	SIP, Gurin’s Global QOL question, SPS, GAF, PANSS, AIMS; HAS, DAI, QLS, WCST	Quality of life predictably influenced by the severity of symptoms, side-effects, cognitive deficits, and dose of antipsychotic medication. The reliability of patients’ reports was not materially affected by these factors.	
4.	Rocca et al. (2015), Italy [40]	Observational, cross-sectional	OUT	323 SCH	DSM-IV-TR		To identify different profiles of functioning by using the QLS and to assess factors associated with best profile membership.	CGI-S, PANSS, CDSS, GAF, Scale for the Assessment of Unawareness of Mental Disorder, QLS	Given three different clusters, being employed and receiving SGAs were associated with a twofold to threefold increased “risk” of “good” cluster affiliation.	Y
5.	Hou et al. (2016), China [41]	Observational, cross-sectional	OUT	607 SCH	ICD-10		Frequency of sexual dysfunction in patients with schizophrenia and impact on QoL.	PRS, SAS, MADRS, ASEX, SF-12	Female gender, being single, older age, and use of first-generation antipsychotics were independently and significantly associated with more sexual dysfunction, which was not associated with lower QOL.	
6.	Medici et al. (2016), Denmark [42]	Observational cross-sectional	OUT	82 SCH	ICD-10		Relation between QOL and illness duration, adjusted daily doses (ADDs) of antipsychotics, body mass index (BMI), waist circumference, and smoking.	WHOQOL-BREF	Lower QOL was associated with high BMI, low adjusted daily doses of antipsychotics, and smoking in first-ever diagnosed patients, and with high BMI and short illness duration in long-term ill patients. A higher daily dose of antipsychotics was weakly associated with higher physical and social QOL and significantly associated with higher environmental QOL among first-ever diagnosed patients.	
7.	Kelin et al. (2011), Australia [37]	Observational, longitudinal	OUT	406 SCH	DSM-IV DSM-IV-TR		To assess patients with schizophrenia at risk of nonadherence who switched to depot and to oral antipsychotics.	PANSS, CGI-S, DAI-10, EQ-5D, SF-12	Patients rated their quality of life and level of functioning as low at study entry and higher by 12 months or endpoint.	
8.	Sungur et al. (2011), Turkey [38]	RCT	OUT	100 SCH	DSM-IV		This study used repeated outcome measures over a 2-year period to compare the clinical and social benefits of routine schizophrenia treatment (OCM) with those of evidence-based pharmacological and psychosocial treatment strategies (RCM).	BPRS, SAPS, SANS, PANSS, HAM-D, Mental Functions impairment scale, DAI, GCS, CAN, QoL	There was a significant improvement in QoL over the 24 months in the RCM group.	
9.	Caqueo-Urìzar et al. (2020), multi-site [44]	Observational transversal ex post facto retrospective	OUT	253 SCH	ICD-10		Adherence to treatment and QoL.		Significant association between treatment adherence and QoL (S-QoL-18 index: β = 0.26, *p* = 0.004; self-esteem: β = 0.37, *p* = 0.000; sentimental life: β = 0.20, *p* = 0.033).	
10.	Cortesi et al. (2013), Italy [39]	Observational, longitudinal	OUT	637 SCH, SPH	DSM-IV, PANSS		Persistence, compliance, costs, and Health-Related Quality-of-Life (HRQoL) in young patients (aged 18–40) suffering from schizophrenia and assuming antipsychotics, comparing naïve (first users) and non-naïve.	CGI-S, GAF, EQ-5D, SF-36naïve-30	Naïve patients had an average higher improvement than the non-naïve, statistically significant in the SF-36 (physical and mental domains). Among the non-naïve patients, significant improvements were found in the CGI-S, GAF, PANSS, and EQ-5D VAS mean scores.	
11.	Vrbova et al. (2017), Czech Republic [43]	Observational, cross-sectional	OUT	52 PD	DSM-5 ICD-10	Antipsychotics in the range of advised therapeutic dose (mean 5.59 ± 3.84 mg dose of risperidone according to antipsychotic index)	Correlation between QoL, self-stigma, hope, and clinical/psychopathological variables.	Q-LES-Q-SUM	At the logistic regression, Q-LES-Q-SUM negatively correlated with the antipsychotic index	

IN: inpatients; OUT: outpatients. SCH: schizophrenia; SPH: schizophreniform disorder; SAD: schizoaffective disorder; DD: delusional disorder; BPD: brief psychotic disorder; FEP: First-Episode Psychosis; PD: Psychotic Disorder. QoL: Quality of Life. RCT: randomized controlled trial. Imp QoL: Improvement in quality of life. Diagnostic and Outcome Assessment tools: AIMS: Abnormal Involuntary Movement Scale; ASEX: Arizona Sexual Experience Scale; BPRS: Brief Psychiatric Rating Scale; CDSS: Calgary Depression Scale for Schizophrenia; CGI: Clinical Global Impression; DAI: Drug Attitude Inventory; DSM: Diagnostic and Statistical Manual of Mental Disorders; GAF: Global Assessment of Functioning; HAM-D: Hamilton Rating Scale for Depression; HAS: Hillside Akathisia scale; ICD-10: International Classification of Diseases 10th Revision; IS: Insight Scale; PANSS: Positive and Negative Syndrome Scale; Q-LES-Q: Quality-of-Life Enjoyment and Satisfaction Questionnaire; QLS: Quality-of-Life Scale; SAS: Simpson Angus Scale; SF: Short-Form Health Survey; SIP: Sickness impact profile; SPS: Social Performance Schedule; WCST: Wisconsin Card Sorting Test; WHOQOL: World Health Organization Quality-of-Life assessment.

**Table 4:** Antipsychotic polytherapy (N = 2).

	Author (Year), Country	Study Design	Setting	Sample (N, Diagnosis)	Diagnostic Assessment	Pharmacological Treatment	Outcome	Outcome Assessment	Results	Imp Qol
1.	Hou et al. (2016), China [46]	Observational, cross-sectional	OUT	623 SCH		APP	Impact of antipsychotic polypharmacy on QoL.		Patients on APP were more likely to receive SGAs and anticholinergics, had fewer hospitalizations, younger age of onset, and higher doses of antipsychotics. No significant differences between the FGA and SGA groups in any of the QOL domains.	y
2.	Li et al. (2015), China [45]	Observational, cross-sectional	IN/OUT	4239 SCH	DSM.IV ICD-10	APP: two or more antipsychotics	Use, demographic, clinical correlates, treatment satisfaction, and quality of life in clinically stable patients with schizophrenia prescribed with antipsychotic polypharmacy treatment.	CGI-S, TESS, SF-12	Lower satisfaction with treatment, higher QOL in the mental domain, younger age of onset, more side-effects, higher doses of antipsychotics were observed among schizophrenia patients with APP treatment.	y

IN: inpatients; OUT: outpatients. SCH: schizophrenia; SPH: schizophreniform disorder; SAD: schizoaffective disorder; DD: delusional disorder; BPD: brief psychotic disorder; FEP: First-Episode Psychosis; PD: Psychotic Disorder. QoL: Quality of Life. RCT: randomized controlled trial. Imp QoL: Improvement in quality of life. Diagnostic and Outcome Assessment tools: CGI: Clinical Global Impression; DSM: Diagnostic and Statistical Manual of Mental Disorders; ICD-10: International Classification of Diseases 10th Revision; SF: Short-Form Health Survey, TESS: Emergent Symptom Scale.

**Table 5:** Studies focusing on oral and depot/LAI formulation and quality of life (N = 4).

	Author (Year), Country	Study Design	Setting	Sample (N, Diagnosis)	Diagnostic Assessment	Pharmacological Treatment	Outcome	Outcome Assessment	Results	Imp Qol
1.	Novick et al. (2012), USA [112]	Observational, longitudinal	OUT	10972 SCH		Olanzapine, Risperidone, Quetiapine, Amilsulpride, Clozapine, Oral typical, Depot typical	12-month outcomes associated with naturalistic antipsychotic treatment	CGI, EQ-5D	Patients in all cohorts except the clozapine cohort had a lower increase in EQ-5D VAS at 12 months compared with olanzapine.	Y
2.	Lambert et al. (2011), Germany [113]	Observational, longitudinal		2224 SCH	DSM-IV	SGA monotherapy, FGA monotherapy (oral and long-acting), combination therapy, no AP	Difference between SGA and FGA in subjects well-being	SWN-K	Small but clinically relevant superiority of SGAs over FGAs in subjective well-being.	y
3.	Alonso et al. (2009), Spain [114]	Observational, longitudinal	OUT	9340 SCH		Olanzapine, risperidone, quetiapine, amisulpride, clozapine, oral typical antipsychotics, and depot typical antipsychotics	Association between continuous antipsychotic use and health-related quality of life (HRQL)	EuroQol-5D, CGI	Continuous antipsychotic treatment is associated with important HRQL benefits at 3 years, most of which occurs during the first 6 months.	y
4.	Sugawara et al. (2019), Japan [115]	Observational, cross-sectional	IN	159 SCH, SAD	DSM-IV	LAI, oral antipsychotics	Symptom severity, side-effects, functioning, QoL, self-esteem	SF-36	No significant differences in SF-36 scores between the two subgroups.	n

IN: inpatients; OUT: outpatients. SCH: schizophrenia; SPH: schizophreniform disorder; SAD: schizoaffective disorder; DD: delusional disorder; BPD: brief psychotic disorder; FEP: First-Episode Psychosis; PD: Psychotic Disorder. QoL: Quality of Life. RCT: randomized controlled trial. Imp QoL: Improvement in quality of life. Diagnostic and Outcome Assessment tools: CGI: Clinical Global Impression; DSM: Diagnostic and Statistical Manual of Mental Disorders; ICD-10: International Classification of Diseases 10th Revision; SF: Short-Form Health Survey; SWN: Subjective Well-Being under Neuroleptic scale.

## 4. Discussion

The present review aims to evaluate the role of antipsychotic medications on the levels of quality of life in patients with schizophrenia spectrum disorders. In particular, quality of life represents a key component of recovery, which is achieved by relatively few subjects with schizophrenia. As recently stated by international guidelines [158], improving and fostering patients’ personal and social functioning in the acute phase of treatment, and continuing to support functioning in the maintenance phase, should be among the major goals of the personalized treatment developed for people with schizophrenia.

The main findings of our review include the following: (1) quality of life is usually considered a secondary outcome of trials on the efficacy and effectiveness of drugs; (2) second- and third-generation antipsychotics have a positive effect on quality of life; and (3) long-acting injectable antipsychotics are associated with a more stable improvement in quality of life and with a good safety and tolerability profile (Table 6).

Regarding the first finding, quality of life was a secondary endpoint in all included studies. This should be due to the fact that quality of life is a complex construct, which can be defined in many different ways [159]. The complexity has also been confirmed by the fact that several assessment tools are used for measuring such dimension. Furthermore, the levels of quality of life are influenced by several contextual and social factors (such as the levels of support from social networks, and the deprivation index in the area in which the patient is living), as well as by clinical variables (such as the severity of cognitive or negative symptoms). Therefore, the boundaries of the construct of “quality of life” should be redefined in order to include this as a primary outcome in efficacy and effectiveness studies.

However, a positive—and quite unexpected—finding is that quality of life has been mentioned among studies’ outcomes since 1990, starting with the study by Meltzer et al. [73]. This is probably due to the fact that quality of life has always been considered an important aspect of patients’ outcome, which has a significant impact on the levels of personal functioning as well as on relapse and hospitalization rates.

When a second- or third-generation antipsychotic is used, the quality of life improves. It is likely that the better tolerability profile of these drugs compared to first-generation antipsychotics is crucial in the assessment of patients’ quality of life [160,161,162,163].

Finally, the use of long-acting injectable antipsychotics was associated with a stable improvement in the levels of quality of life. This effect should be due to the several pharmacokinetic advantages introduced by LAI drugs, which allow for a more stable blood concentration of the drug, with lower rates of side-effects and a better long-term compliance with the treatment.

It should be noted that only one study carried out in 90s’ analyzed the impact of neuroleptics on the levels of quality of life of patients with schizophrenia. These data confirm that the quality of life for many decades has not been considered as a relevant clinical dimension for the recovery process of patients with schizophrenia, whereas the reduction in the severity of positive symptoms has been prioritized.

The impact of psychotropic medications on levels of quality of life in people with severe mental disorders has been extensively evaluated in samples of patients suffering from bipolar disorder and treated with lithium [164] or with valproic acid [165] or in patients suffering from major depressive disorder [166]. To date, the key role of quality of life in the long-term recovery journey has been widely accepted for all patients suffering from severe mental disorders, but it has been more studied in samples of patients with affective disorders. This represents a relevant unmet need in the management plan of patients with schizophrenia, which should be appropriately filled in. Therefore, the results of the present systematic review should be useful to inform further studies evaluating the long-term efficacy and effectiveness of antipsychotic drugs on clinical dimensions, such as quality of life, which has been overlooked and neglected for many years.

Our systematic review has some limitations, which should be acknowledged. Firstly, in more than 50% of studies, patients were recruited in outpatient settings, which might be affected by a less severe type of the disorder. However, it should be considered that quality of life is a multidimensional construct influenced by several clinical, social, and environmental variables and is strongly dependent on the clinical situation of the moment. Moreover, the search strategy has only been limited to studies including adult patients aged over 18 years. This methodological choice was due to the fact that the presentation of schizophrenia and its treatment in late childhood and/or adolescence can have different clinical and psychosocial characteristics, which are usually assessed through specific assessment tools, specifically validated for the young population. Therefore, a further literature search with a specific focus on patients with a childhood/adolescent onset of schizophrenia should be performed, and the results could be useful to support the development of youth mental health services [167,168,169].

Another limitation is related to the heterogeneity of tools adopted for measuring quality of life. Although all included assessment instruments were validated and reliable, they present specific differences in catching the subtle, different components of quality of life.

## 5. Conclusions

The present systematic review confirms that quality of life represents a central element for selecting the appropriate treatment for people with schizophrenia. Within the unmet clinical needs that directly impact the quality of life of these patients, the availability of new treatments with a better tolerability profile, a proven effectiveness on patients’ cognitive and social functioning, and a more stable blood concentration might represent the appropriate strategies [170].

## Figures and Tables

**Table 6:** Key messages.

(1) quality of life has been a neglected and overlooked dimension in the management plan of patients with schizophrenia
(2) quality of life has been usually considered a secondary outcome of trials on efficacy and effectiveness of drugs
(3) second- and third-generation antipsychotics have a relevant impact on quality of life
(4) long-acting injectable antipsychotics are associated with a more stable improvement in quality of life and with good safety and tolerability profile

## Data Availability

Data available on request.

## Supplementary Materials

The following supporting information can be downloaded at: https://www.mdpi.com/article/10.3390/brainsci13111577/s1, Table S1. Risk of bias assessment in randomized clinical trials (RCTs). Table S2. Risk of bias assessment in non-randomized studies of intervention (NRSI).
