# Supplementary material for: A Systematic Review on the Effectiveness of Antipsychotic Drugs on the Quality of Life of Patients with Schizophrenia
Source: Brain Sci. 2023 Nov 10;13(11):1577. doi: 10.3390/brainsci13111577 (PMC10669728; doi:10.3390/brainsci13111577)
Supplement: Supplementary file 1 [file brainsci-13-01577-s001.zip › brainsci-2689321-SI.pdf]

Supplementary Table S1. *Risk of bias assessment in randomized clinical trials (RCTs).*

First Author (year of publication)	Randomization process	Deviation from intended intervention	Missing outcome data	Measurement of the outcome	Selection of the reported results	Overall risk of bias
**Awad et al. (1997a)**	Low	Low	Moderate	Moderate	Moderate	**Moderate**
**Hamilton et al. (1998)**	Low	Low	Low	Low	Low	**Low**
**Awad et al. (2014)**	Moderate	Low	Low	Low	Low	**Moderate**
**Naber et al. (2013)**	Moderate	Low	Moderate	Low	Low	**Moderate**
**Lin et al. (2013)**	Moderate	Moderate	Low	Moderate	Moderate	**Moderate**
**Kusumi et al. (2012)**	Low	Moderate	Low	Low	Low	**Moderate**
**Ishigooka et al. (2021)**	Low	Moderate	High	High	Moderate	**High**
**Iyo et al. (2021)**	Moderate	Moderate	High	High	Moderate	**High**
**Veselinovic et al. (2019)**	Low	Low	Low	Moderate	Moderate	**Moderate**
**Gründer et al. (2016)**	Low	Moderate	Moderate	Moderate	High	**High**
**Fervaha et al. (2014)**	Moderate	Low	Low	Low	Low	**Moderate**
**Nilsen et al. (2012)**	Low	Low	Low	Low	Low	**Low**
**Naber et al. (2015)**	Low	Low	Low	Low	Low	**Low**
**Rouillon et al. (2013)**	Low	Low	Moderate	Low	Moderate	**Moderate**
**Ascher-Svanum et al. (2014)**	Moderate	Low	Moderate	Low	Low	**Moderate**

**Ascher-Svanum et al. (2011)**	Low	Low	Moderate	Low	Low	**Moderate**
**Witte et al. (2012)**	Low	Low	Low	Low	Low	**Low**
**Potkin et al. (2017)**	Low	Low	Low	Low	Low	**Low**
**Schreiner et al. (2015)**	Low	Low	Low	Low	Low	**Low**
**Leatherman et al. (2014)**	Low	Low	Low	Moderate	Moderate	**Moderate**
**Isitt et al. (2016)**	Low	Low	Low	Low	Low	**Low**
**Sangur et al (2011)**	Low	Low	Moderate	Moderate	Moderate	**Moderate**

Supplementary Table S2. *Risk of bias assessment in non-randomized studies of intervention (NRSI).*

First Author (year of publication)	Type of study	*Pre-intervention domains*		*At-intervention domain*	*Post-intervention domains*				Overall risk of bias
		**Confounding bias**	**Selection bias**	**Information bias**	**Confounding bias**	**Selection bias**	**Information bias**	**Reporting bias**	
**Chiliza et al. (2015)**	Longitudinal	Low	High	High	Low	High	High	High	**High**
**Schmauss et al. (2010)**	Prospective, multicentre, open-label, non-randomised trial	Low	Moderate	Moderate	High	Moderate	Moderate	Low	**High**
**Lambert et al. (2010)**	Longitudinal	High	Moderate	Moderate	Moderate	High	Moderate	Moderate	**High**
**Pietrini et al. (2016)**	Longitudinal	Moderate	Moderate	Low	Moderate	Moderate	Moderate	Moderate	**Moderate**
**Browne et al. (1998)**	Cross-sectional	High	Moderate	Low	Moderate	Moderate	Moderate	Low	**High**
**Awad et al. (1997)**	Cross-sectional								
**Voruganti et al. (1998)**	Longitudinal	Moderate	Moderate	Low	Moderate	Moderate	Moderate	Low	**Moderate**
**Rocca et al. (2015)**	Cross-sectional	Moderate	Moderate	Low	Moderate	High	Moderate	Moderate	**Moderate**
**Hou et al. (2015a)**	Cross-sectional	Moderate	Low	Low	Moderate	Moderate	Moderate	Moderate	**Moderate**
**Medici (2016) et al.**	Cross-sectional	Moderate	Moderate	High	Moderate	High	High	Moderate	**High**
**Kelin et al. (2011)**	Longitudinal	Moderate	Moderate	Moderate	Moderate	Moderate	Moderate	Low	**Moderate**
**Caqueo-Urìzar et al. (2020)**	Retrospective	High	Low	Low	High	Low	Low	Low	**Moderate**

**Cortesi et al. (2013)**	Longitudinal	High	High	Moderate	High	High	Moderate	Low	**HIgh**
**Vrbova et al. (2017)**	Cross-sectional	Moderate	Moderate	Low	Moderate	Moderate	Low	Low	**Moderate**
**Hou et al. (2016)**	Cross-sectional	High	High	Moderate	High	High	Moderate	Moderate	**High**
**Li et al., (2015)**	Cross-sectional	High	High	Moderate	High	High	Moderate	Moderate	**High**
**Novick et al., (2012)**	Longitudinal	High	Low	High	High	Low	High	High	**High**
**Lambert et al., (2011)**	Longitudinal	High	Low	High	High	Low	High	High	**High**
**Alonso et al. (2009)**	Longitudinal	High	Low	High	High	Low	High	High	**High**
**Sugawaea et al., (2019)**	Cross-sectional	High	Moderate	High	High	Moderate	High	High	**High**

**Morss (1994)**	Observational	Low	Low	Low	Low	Low	Low	Low	**Low**
**Bobes (1998)**	Longitudinal	Moderate	Moderate	Moderate	Moderate	Low	Moderate	Moderate	**Moderate**
**Galletly (2019)**	Cross-sectional	High	Moderate	Moderate	High	Moderate	Moderate	Moderate	**High**
**Meltzer (1990)**	Longitudinal	Moderate	Low	Moderate	Moderate	Low	Moderate	Moderate	**Moderate**
**Dima (2015)**	Observational	Moderate	High	Moderate	Moderate	High	Moderate	Moderate	**High**
**Li (2015)**	Cross-sectional	Moderate	Low	Moderate	Moderate	Low	Moderate	Moderate	**Moderate**
**Araujo (2015)**	Cross-sectional	Moderate	Low	Moderate	Moderate	Moderate	Moderate	Moderate	**Moderate**
**Hashimoto (2015)**	Longitudinal	High	High	High	High	High	High	High	**High**

**Montgomery (2015)**	Prospective Observational	High	High	High	High	High	High	High	**High**
**Araujo (2014)**	Cross-sectional	Moderate	Low	Moderate	Moderate	Moderate	Low	Moderate	**Moderate**
**Schreiner (2014)**	Naturalistic	NA	NA	NA	NA	High	Moderate	High	**High**
**Gattaz (2014)**	Longitudinal	High	Moderate	Moderate	Moderate	High	Moderate	Moderate	**High**
**Gutiérrez Fraile (2013)**	Longitudinal	High	High	Moderate	High	Moderate	Moderate	Low	**High**
**Yeh (2013)**	Longitudinal	High	High	Moderate	High	Moderate	Moderate	Moderate	**High**
**Bervoets (2012)**	Longitudinal	High	Moderate	Moderate	High	Moderate	Moderate	Moderate	**High**
**Peuskesn (2012)**	Prospective Observational	Moderate	High	Moderate	Moderate	High	Moderate	Low	**High**
**Ye (2011)**	Prospective Observational	High	Moderate	Low	High	Moderate	Low	Low	**High**
**Ye (2012)**	Prospective Observational	High	High	Moderate	High	High	Moderate	Low	**High**
**Liu-Seifert (2012)**	Longitudinal	High	High	Moderate	High	High	Moderate	Moderate	**High**
**Mahmoud (2011)**	Longitudinal	High	Moderate	Moderate	High	Moderate	High	High	**High**
**Ming-Hong Hsieh (2010)**	Longitudinal	Moderate	High	Moderate	Moderate	High	Moderate	Low	**High**
**Ching-Hua Lin (2010)**	Longitudinal	Moderate	High	Moderate	Moderate	High	High	Low	
**Larsen & Gerlach (1996)**	Cross-sectional	High	High	High	Moderate	High	High	Moderate	High
**Cheng-TaLi et al. (2010)**	Cross-sectional	Moderate	Moderate	Moderate	Low	Low	Low	Low	Moderate

**Peuskens et al. (2012b)**	Longitudinal	Low	Low	Low	Low	Low	Low	Low	Low
**Lee et al. (2014)**	Longitudinal	Low	Low	Moderate	High	Moderate	Low	Low	Moderate
**Chiliza et al.(2015)**	Longitudinal	Moderate	Moderate	Moderate	Low	Moderate	Low	Low	Moderate
**Niolu et al. (2015)**	Longitudinal	Moderate	Moderate	Low	Low	Low	Low	Low	Low
**Pietrini et al. (2015)**	Longitudinal	Moderate	Low	Low	Low	Low	Low	Low	Low
**Naber et al. (2017)**	Longitudinal	Low	Low	Low	Low	Low	Low	Low	Low
**Llorca et al. (2018)**	Longitudinal	Low	Moderate	Low	Low	Low	Low	Low	Low
**Aykut et al. (2019)**	Cross-sectional	Moderate	Moderate	Low	Moderate	Low	Low	Low	Moderate
**Giordano et al. (2020)**	Longitudinal	Low	Moderate	Low	Low	Low	Moderate	Moderate	Low
**Phahladira et al. (2020)**	Longitudinal	Moderate	Low	Low	Low	Low	Low	Low	Low
**Pietrini et al. (2015)**	Longitudinal	Moderate	Low	Low	Low	Moderate	Low	Low	Moderate
**McEvoy et al. (2021)**	Post-hoc analysis	Low	Low	Moderate	Moderate	Low	Low	Low	Moderate
**Aitchison (2011)**	Longitudinal	High	High	High	High	High	High	High	**High**
**Kim & Kim (2009)**	Cross-sectional	Moderate	Low	Low	Moderate	Moderate	Moderate	Moderate	**Moderate**
**Popolo (2010)**	Longitudinal	High	High	Hight	Moderate	moderate	High	Moderate	**High**
**Verma (2020)**	Longitudinal	Moderate	Moderate	Moderate	Moderate	Moderate	Moderate	Moderate	**Moderate**

**Lee (2016)**	Cross-sectional	Moderate	Moderate	Low	Moderate	High	Moderate	Moderate	**Moderate**
**Awad (2016)**	Longitudinal	Moderate	Moderate	Moderate	Moderate	Low	Moderate	Moderate	**Moderate**
**Kao (2011)**	Cross-sectional	High	Hight	Moderate	Moderate	Moderate	Moderate	Moderate	**Moderate**
**Hasan (2018)**	Cross-sectional	Moderate	Moderate	Moderate	Moderate	Moderate	Moderate	Moderate	**Moderate**
**Hou (2015b)**	Cross-sectional	Moderate	Moderate	Low	Moderate	Moderate	Moderate	Moderate	**Moderate**
**Shrivastava (2012)**	Cross-sectional	Low	Moderate	Low	Moderate	Moderate	Moderate	Hight	**High**
**Kim (2014)**	Longitudinal, cross-sectional	Moderate	Low	Low	Moderate	Low	Moderate	Moderate	**Moderate**
**Huang (2013)**	Longitudinal	High	Hight	Moderate	Moderate	High	Moderate	High	**High**
**Melo Chaves (2013)**	Cross-sectional	Moderate	Moderate	Low	Moderate	Moderate	Moderate	Moderate	**Moderate**
**Ye (2014)**	Longitudinal	High	Moderate	High	High	Moderate	Moderate	Moderate	**Moderate**
**Kilian (2012)**	Longitudinal	Moderate	Moderate	Low	Moderate	Moderate	Moderate	Moderate	**Moderate**
**Adrianzen (2010)**	Longitudinal	Low	Moderate	Moderate	Moderate	High	Moderate	Moderate	**Moderate**
